# Impact of anesthesia modalities on functional outcome of mechanical thrombectomy in patients with acute ischemic stroke: a subgroup analysis of DIRECT-MT trial

**DOI:** 10.1186/s40001-023-01171-x

**Published:** 2023-07-10

**Authors:** Zifu Li, Hongyu Ma, Binben Li, Lei Zhang, Yongwei Zhang, Pengfei Xing, Yongxin Zhang, Xiaoxi Zhang, Yu Zhou, Qinghai Huang, Qiang Li, Qiao Zuo, Xiaofei Ye, Jianmin Liu, Adnan I. Qureshi, Wenhuo Chen, Pengfei Yang, DIRECT-MT Investigators

**Affiliations:** 1 Neurovascular Center, Changhai Hospital, Naval Medical University, Shanghai, China; 2 Department of Anesthesiology, Changhai Hospital, Naval Medical University, Shanghai, China; 3 Zeenat Qureshi Stroke Institute and Department of Neurology, University of Missouri, Columbia, MO USA; 4 Department of Neurology, Municipal Hospital of Zhangzhou, Zhangzhou, Fujian Province China

**Keywords:** Mechanical thrombectomy, Acute ischemic stroke, Anesthesia, Outcome

## Abstract

**Background:**

This subgroup analysis of Direct Intraarterial Thrombectomy in Order to Revascularize Acute Ischemic Stroke Patients with Large Vessel Occlusion Efficiently in Chinese Tertiary Hospitals Multicenter Randomized Clinical Trial (DIRECT-MT) aimed to investigate the influence of anesthesia modalities on the outcomes of endovascular treatment.

**Methods:**

Patients were divided into two groups by receiving general anesthesia (GA) or non-general anesthesia (non-GA). The primary outcome was assessed by the between-group difference in the distribution of the modified Rankin Scale (mRS) at 90 days, estimated using the adjusted common odds ratio (acOR) by multivariable ordinal regression. Differences in workflow efficiency, procedural complication, and safety outcomes were analyzed.

**Results:**

Totally 636 patients were enrolled (207 for GA and 429 for non-GA groups). There was no significant shift in the mRS distribution at 90 days between the two groups (acOR, 1.093). The median time from randomization to reperfusion was significantly longer in GA group (116 vs. 93 min, *P* < 0.0001). Patients in non-GA group were associated with a significantly lower NIHSS score at early stages (24 h, 11 vs 15; 5–7 days or discharge, 6.5 vs 10). The rate of severe manipulation-related complication did not differ significantly between GA and non-GA groups (0.97% vs 3.26%; *P* = 0.08). There are no differences in the rate of mortality and intracranial hemorrhage.

**Conclusions:**

In the subgroup analysis of DIRECT-MT, we found no significant difference in the functional outcome at 90 days between general anesthesia and non-general anesthesia, despite the workflow time being significantly delayed for patients with general anesthesia.

*Clinical trail registration* clinicaltrials.gov Identifier: NCT03469206.

**Supplementary Information:**

The online version contains supplementary material available at 10.1186/s40001-023-01171-x.

## Introduction

Mechanical thrombectomy (MT) is increasingly widespread in the treatment of acute ischemic stroke (AIS) secondary to large vessel occlusion (LVO) [1]. However, the optimal anesthesia modality for performing mechanical thrombectomy remains controversial.

General anesthesia (GA) eliminates patient incompliance, reduces pain associated with procedures, and facilitates endovascular procedures such as roadmap navigation, whereas the GA approach potentially increases the risk of periprocedural hemodynamic fluctuation and respiratory complications, and delays workflow efficiency [2–5]. In contrast, the non-general anesthesia (non-GA) approach consisting of either local anesthesia (LA) or conscious sedation (CS) reduces the time from onset to reperfusion, and avoids hemodynamic compromise, but may increase technical complications [3, 6, 7]. So far, the consensus in clinical outcomes associated with anesthesia type has not been well established.

Several studies have suggested that the rates of mortality or disability were lower with non-GA in AIS patients undergoing MT [8, 9]. However, randomized clinical trials (RCTs) have not identified any differences in the rates of mortality or disability [10]. In the meantime, different target populations, workflow patterns, and other factors also contribute to the discrepancy. DIRECT-MT (Direct Intraarterial Thrombectomy in Order to Revascularize Acute Ischemic Stroke Patients with Large Vessel Occlusion Efficiently in Chinese Tertiary Hospitals Multicenter Randomized Clinical Trial) is a clinical trial targeting AIS patients in China who underwent mechanical thrombectomy in a different workflow pattern. The primary outcome of this clinical trial showed endovascular thrombectomy alone was noninferior to thrombectomy preceded by alteplase administration [11]. To provide additional data in east Asian populations, we studied the effect of different anesthesia types on early and late functional outcomes by performing a subgroup analysis of the trial.

## Methods

### Study design and patients

DIRECT-MT was a multicenter phase III prospective randomized clinical trial with open-label treatment and blinded outcome assessment to determine whether direct mechanical thrombectomy (MT) was noninferior to combined intravenous thrombolysis plus MT in patients with AIS due to an anterior circulation large vessel occlusion treatable within 4.5 h after symptom onset. The trial design, patient inclusion and exclusion criteria, treatment protocol, and final results have been previously published [11, 12].

This subgroup analysis was performed based on the intention-to-treat population of DIRECT-MT trial. Patients with an anesthesia record were enrolled and divided into GA group and non-GA group by whether receiving treatment under GA plus mechanical ventilation or non-GA including local anesthesia and conscious sedation. The choice of GA or non-GA was at the discretion of the participating centers, and the data were prospectively recorded. Those who were converted from non-GA to GA during MT were scored as non-GA according to the intention-to-treat principle. The workflow time intervals including the anesthesia procedure were also recorded. This study was approved by hospital committees on ethics of medicine and the research board of all participating centers. The article of this study was prepared following CONSORT reporting guidelines.

### Outcomes

The primary outcome was measured via modified Rankin Score (mRS) (range 0–6; 6 indicates death) at 90 days post randomization. The mRS score was further categorized as an excellent functional outcome (mRS of 0–1), a favorable functional outcome (mRS of 0–2), and a moderate functional outcome (mRS of 0–3). Barthel index was also ascertained at 90 days. The clinical secondary outcomes were the improvement of National Institutes of Health Stroke Scale score (NIHSS) at 24 h and 5–7 days or discharge post-procedural 5–7 days after randomization. The procedural secondary outcomes included angiographic recanalization measured via the extended thrombolysis in cerebral infarction (eTICI) score (range 0–3), total attempts of thrombectomy, and outcome lesion volume on computed tomography (CT).

Safety outcomes were death, asymptomatic intracranial hemorrhages and symptomatic intracranial hemorrhages according to the Heidelberg criteria [13], cerebral infarction in new vascular territory at 5 to 7 days, and mortality within 90 days. Technical complications included any procedural complications, vessel dissection, contrast extravasation, and embolization in new cerebrovascular territories. Severe manipulation-related complications, including contrast extravasation and vessel perforation, were defined as any contrast leakage visible on dynamic angiography due to manipulation related to the MT procedure.

The outcomes of patients were independently analyzed. The functional outcome was evaluated by an independent outcome assessment committee and the adverse event was judged by the adverse event committee. Radiological outcomes were evaluated by an independent imaging core laboratory.

### Statistical analysis

Data were presented as median (interquartile range [IQR]) or as percentages. The normality of distributions was assessed using histograms and Shapiro–Wilk test. The baseline characteristics between GA and non-GA patients were compared using the *χ*^2^ test for categorical variables, the Cochran–Mantel–Haenszel test for stratified categorical data, and Wilcoxon rank-sum test for non-Gaussian distributions. For the relation between anesthesia modality and functional outcome, multivariable ordinal regression analysis was performed to calculate the adjusted common odds ratio for a shift in a direction towards a better functional outcome on the mRS between GA and non-GA group. The regression model was adjusted for age, sex, medical history, NIHSS score, prestroke mRS, ASPECTS, bridging therapy, etiology, and occlusion site in order to reduce the potential baseline imbalances between the two groups. The logistic regression model was used to calculate the adjusted and unadjusted odds ratio (OR) and 95% CI for primary and secondary outcomes. The subgroup analysis of favorable functional outcome (mRS 0–2) was conducted with multivariable ordinal regression based on the dichotomized variables: (1)whether the patient was over-aged, age 80 years or younger vs. older than 80 years [14]; (2)severe or non-severe neurological impairment, baseline NIHSS score less than or equal to 14 vs. greater than 14 [15]; (3) occlusion location, occlusion sites including M1, M2, and ICA; (4)early sign of poor outcomes by imaging ischemia detection, Alberta Stroke Program Early CT Score (ASPECTS) less than 8 vs. 8 to 10 [16]; (5) combination therapy or endovascular alone, with or without using alteplase (rt-PA); (6) whether the endovascular treatment was performed at the very early stage, dichotomized stroke onset to triage not more than 120 min vs. more than 120min [17].

The safety, procedural outcomes, and the rate of technical complications were compared with Chi-square test or adjusted Chi-square test. Logistic regression models were used to estimate the difference in safety outcomes and technical complications between the two groups. The results were estimated as risk differences, and risk ratios (RRs) with 95% confidence intervals (CIs). The subgroup analysis was performed using the SAS (version 9.4) and R (version 4.1.3) software packages. A level of *P* < 0.05 was accepted as statistically significant.

## Results

**Fig. 1:**
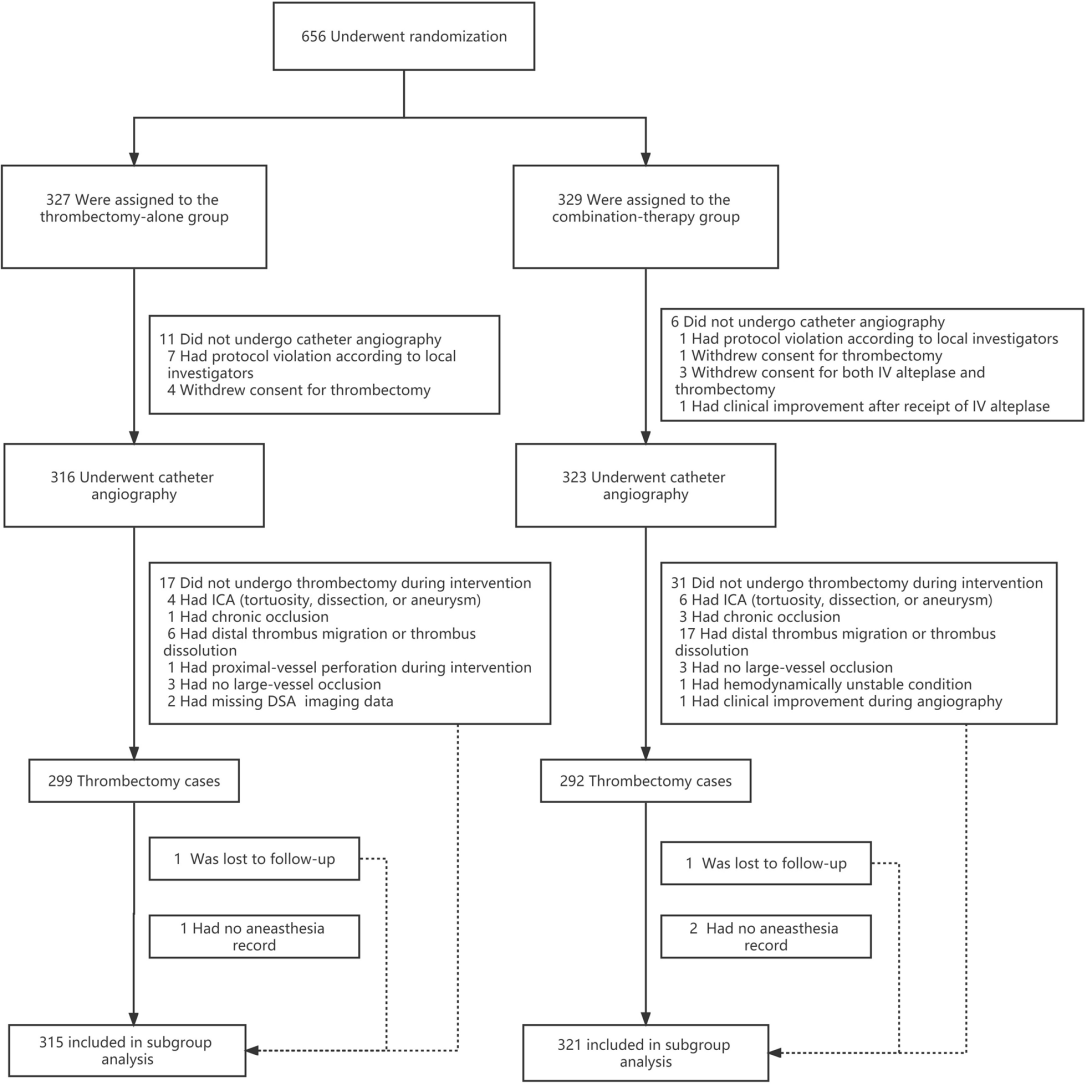
The flowchart of patients selection for this subgroup analysis

The trial profile is shown in Fig. 1. Of the 656 patients enrolled under randomization, 636 patients receiving catheter angiography were included for the subgroup analysis, and the remaining 20 patients were excluded including 17 patients who did not undergo catheter angiography and 3 patients loss of anesthesia record. A total of 299 patients were assigned to undergo endovascular thrombectomy alone and 292 were assigned to receive combination therapy with intravenous alteplase and mechanical thrombectomy. GA and non-GA were used in 207 and 429 patients, respectively.

The baseline characteristics of demographics, medical history, prestroke mRS, stroke etiology, and occlusion site were comparable between the two groups (Table 1). The ASPECTS score in non-GA group was higher than that in GA group (median 9 *vs.* 8, *P* = 0.009). The baseline systolic blood pressure tended to be higher in GA group than that in non-GA group (median 148 vs. 144, *P* = 0.06).

**Table 1:** Baseline characteristics

Characteristics	Non-general anesthesia (N = 429)	General anesthesia (N = 207)	*P Value*
Median age- yr	69.00	71.00	0.21
Male sex- no. (%)	241(56.18)	118(57.00)	0.84
Medical history- no. (%)			
Hypertension	250(58.28)	130(62.80)	0.28
Atrial fibrillation	196(45.69)	98(47.34)	0.70
Diabetes mellitus	77(17.95)	43(20.77)	0.39
Clinical status			
NIHSS score, Median	17.00	17.00	0.47
Baseline SBP, Median	144.00	148.00	0.06
Pre-stroke mRS- no. (%)			0.45
0	392(91.38)	195(94.20)	
1	29(6.76)	7(3.38)	
2	8(1.86)	5(2.42)	
ASPECTS	9.00	8.00	0.009
Bridging therapy- no. (%)			
EVT	210(48.95)	105(50.72)	0.68
EVT + IVT	219(51.05)	102(49.28)	
Etiology- no. (%)			0.66
Cardioembolic	189(44.06)	94(45.41)	
Intracranial atherosclerosis	27(6.29)	18(8.70)	
Undetermined	170(39.63)	76(36.71)	
Occlusion site- no. (%)			0.38
ICA	157(37.12)	66(32.04)	
M1	215(50.83)	119(57.77)	
M2	50(11.82)	21(10.19)	
Workflow intervals- median, min			
Onset to triage	108.00	126.00	0.08
To intravenous alteplase	180.00	190.00	0.10
To randomization	169.00	177.00	0.08
To reperfusion	238.00	262.00	0.0002
Triage to randomization	49.00	49.00	0.695
To intravenous alteplase	60.00	58.00	0.54
To groin puncture	84.00	85.00	0.30
To reperfusion	148.50	169.00	0.0003
Randomization to groin puncture	32.00	36.00	0.09
To reperfusion	93.00	116.00	< 0.0001
Groin puncture to reperfusion	59.00	72.00	0.002

*ASPECTS* Alberta Stroke Program Early CT score; *SBP* systolic blood pressure; *ICA* internal carotid artery; *M* middle cerebral artery; *NIHSS* National Institutes of Health Stroke Scale; *EVT* endovascular treatment; *IVT* intravenous treatment

### Workflow outcomes

The median workflow time intervals in GA group were significantly longer than those in non-GA group in regard to the time interval between triage to reperfusion (148 min vs. 169 min, *p* = 0.0003), and randomization to reperfusion (93 min vs. 116 min, *p* < 0.0001), while the intervals between stroke onset to triage, triage to intravenous alteplase, and hospital admission to groin puncture were comparable between the two groups (108 min vs. 126 min, *p* = 0.08; 60 min vs. 58 min, *p* = 0.54; 84 min vs. 85 min, *p* = 0.30).

### Functional outcome

**Fig. 2:**
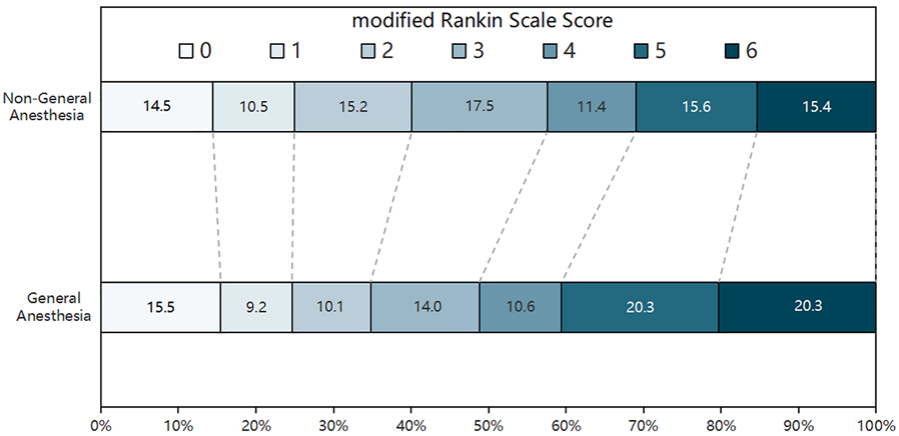
The distribution of modified Rankin Scale (mRS) at 90 days between general anesthesia and non-general anesthesia group

**Fig. 3:**
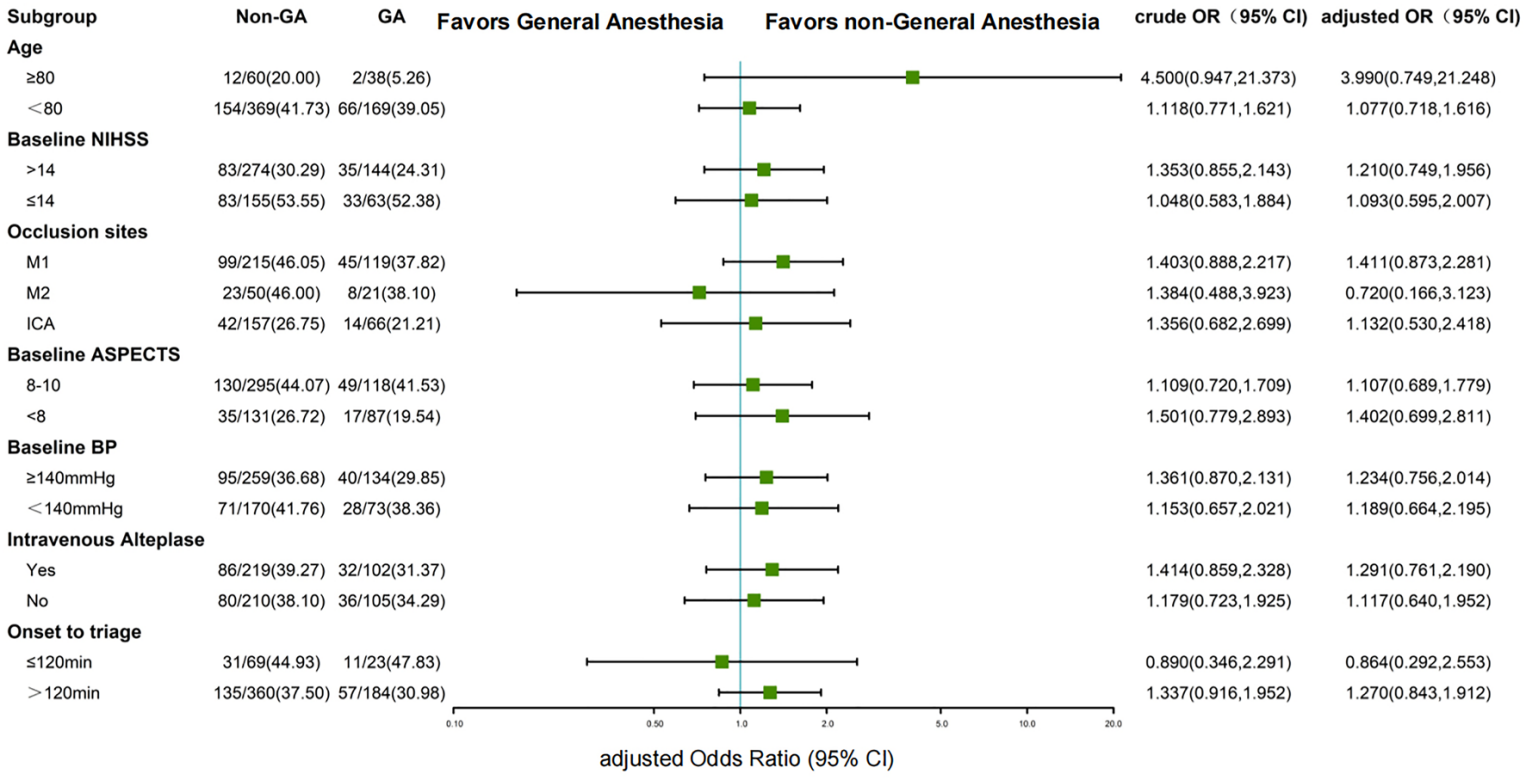
Subgroup analysis for the favorable functional outcome in each dichotomized subgroup. *Non-GA* non-general anesthesia; *GA* general anesthesia; *ICA* internal carotid artery; *M* middle cerebral artery; *Bp* blood pressure

The median mRS at 90 days was 3 (Q1,2; Q3,5) in non-GA group and 4 (Q1,2; Q3,5) in GA group (*P* = 0.10). There was no significant shift in direction of a better outcome in the distribution of the mRS (adjusted cOR, 1.093 [95% CI, 0.807–1.482]) (Fig. 2). The dichotomized results of the score *χ*^2^ test to assess the proportional assumption also showed no significant difference in terms of the excellent functional outcome (mRS 0–1; non-GA *vs.* GA; 23.31% *vs.* 24.15%; P = 0.81) and favorable functional outcome (mRS 0–2; non-GA *vs.* GA; 38.69% *vs.* 32.85%; *P* = 0.15) and better moderate functional outcome in non-GA group (mRS 0–3; non-GA *vs.* GA; 56.18% vs. 47.34% P = 0.04). Moreover, adjusted analysis in logistic regression model showed no significant differences between the two groups in terms of the rates of excellent functional outcome (mRS 0–1; non-GA *vs.* GA; adjusted odds ratio [AOR], 0.776 [95% CI 0.505–1.192]), favorable functional outcome (mRS 0–2; Non-GA *vs.* GA; AOR, 1.108 [95% CI 0.747–1.642]), and moderate functional outcome (mRS 0–3; non-GA *vs.* GA; AOR, 1.297 [95% CI 0.888–1.893]) (Additional file 1: Fig. S1). In the subgroup analyses of favorable functional outcomes, no significant differences between any dichotomized subgroups were identified (Fig. 3).

The early median NIHSS score in non-GA group was significantly lower as compared with that in GA group at 24 h (11 vs. 15; *P* = 0.001) and 5–7 days or discharge (6.5 vs. 10; *P* = 0.046). No difference was observed in the rate of Barthel Index 95 or 100 (non-GA *vs.* GA; AOR, 1.393 [95% CI 0.951–2.040]) (Table 2 and Additional file 1: Fig. S1).

**Table 2:** Primary and secondary outcomes of patients in DIRECT-MT

	Non-general anesthesia	General anesthesia	*P* value
Clinical outcomes			
mRS score at 90 days, median(IQR)	3(2–5)	4(2–5)	0.10
mRS of 0–1 at 90 days, *n* (%)			
0–1	100(23.31)	50(24.15)	0.81
2–6	329(76.69)	157(75.85)	
mRS of 0–2 at 90 days, *n* (%)			
0–2	166(38.69)	68(32.85)	0.15
3–6	263(61.31)	139(67.15)	
mRS of 0–3 at 90 days, *n* (%)			
0–3	241(56.18)	98(47.34)	0.04
4–6	188(43.82)	109(52.66)	
NIHSS score after 24 h, median (IQR)	11(4–19)	15(6–26)	0.001
NIHSS score at 5–7 days or discharge, median (IQR)	6.5(2–16)	10(2–23)	0.046
Barthel Index at 90 days, *n* (%), 95 OR 100	215(50.12)	81(39.13)	0.009
Procedural outcomes			
eTICI score assessed on final angiogram, *n* (%)			
≥ 2b	340(81.34)	169(83.25)	0.56
< 2b	78(18.66)	34(16.75)	
Total attempts of thrombectomy, median (IQR)	2(1–3)	2(1–3)	0.29
Outcome lesion volume on CT, median (IQR)^a^	36.1(9.44,106.40)	38.1(11.33,103.62)	0.98
Safety outcomes			
Death, * n* (%)	73(17.02)	45(21.74)	0.15
Symptomatic intracranial hemorrhage, *n* (%)^b^	25(5.83)	8(3.86)	0.30
Asymptomatic intracranial hemorrhage, *n* (%)	151(35.20)	77(37.20)	0.62
Infarction in new territory at 5–7 days, *n* (%)	17(3.96)	3(1.45)	0.089
Technical complications			
Any procedural complications, *n* (%)	68(15.85)	27(13.04)	0.35
Vessel dissection, *n* (%)	8(1.86)	5(2.42)	0.87
Contrast extravasation, *n* (%) ^c^	14(3.26)	2(0.97)	0.08
Embolization into a new territory, *n* (%)^d^	45(10.49)	20(9.66)	0.75

*mRS* modified Rankin Scale; *IQR* interquartile range; *eTICI* extended Thrombolysis in Cerebral Infarction; *CT* computed tomography

^a^The volume was calculated by the StrokeViewer software at Nicolab in Netherlands

^b^The definition of symptomatic intracranial hemorrhage was according to the Heidelberg criteria

^**c**^Contrast extravasation was defined as contrast leakage on dynamic angiography confirmed by imaging core lab

^d^The definition of embolization in a new territory was defined as the angiographic occlusion in a previously unaffected vascular territory observed on the angiogram

### Procedural and safety outcomes

In terms of procedural outcomes in both groups, no significant differences were observed in the rates of successful reperfusion, total attempts of thrombectomy, and outcome lesion volume on CT. In terms of safety outcomes in both groups, there were also no significant differences in the rates of death, asymptomatic or symptomatic intracranial hemorrhage, and infarction in new territory at 5–7 days between the two groups (Table 2 and Additional file 1: Fig. S2).

### Technical complications

With respect to severe manipulation-related complications, the rate of contrast extravasation on dynamic angiography tends to be higher in non-GA group with 14 cases including 2 cases of vessel perforation, and 2 in GA group during the MT procedure (14:2; 3.26% *vs.* 0.97%; *P* = 0.08) (Table 2). Of 14 cases with contrast extravasation in non-GA group, the affected sites involved M1 in 5 cases, ICA in 7 cases at ICA, and M2 in 2 cases, of whom 10 received rt-PA intravenous thrombolysis. All 14 cases had death or disability (mRS 6,6; mRS 5,4; mRS 4, 1; mRS 3, 2; mRS 2, 1) and low reperfusion score (eTICI 2c, 2; eTICI 2b, 8; eTICI 2a,1; eTICI 0,3). Two cases of contrast extravasation in GA group, respectively, occurred at M1 and ICA, in whom mRS was 3 and 6 at 90 days. No statistical differences in other procedural complications were otherwise observed between the two groups (Table 2 and Additional file 1: Fig. S2).

## Discussion

In the subgroup analysis of DIRECT-MT, no significant difference was observed between GA and non-GA groups on the functional outcome at 90 days, while the early neurological improvement in non-GA group was better than that in GA group in terms of NIHSS score at 24 h and 5–7 days or discharge. Patients in GA group were associated with a significant delay in the workflow time interval but GA was likely to incur fewer severe procedural complications compared with non-GA, although the between-group difference was not significant. The choice of anesthesia patterns was determined by the condition of patients. Non-GA usually is the first choice because of the possible time saving. GA would be performed if the patients cooperated poorly or had high risk of aspiration and troubles in the management of blood pressure and respiration.

Our findings were in line with the result of THRACE trial [18] that GA and non-GA groups did not exhibit significant differences in the mRS distribution. Recent randomized trials regarding anesthesia choices in anterior stroke including Sedation vs Intubation for Endovascular Stroke Treatment (SIESTA), Anesthesia During Stroke (AnStroke), General or Local Anesthesia in Intra Arterial Therapy (GOLIATH), and General Anesthesia versus Sedation for Acute Stroke Treatment (GASS) also did not show worse clinical outcome on 90-day mRS within patients who underwent mechanical thrombectomy with GA [10, 19–21]. Choice of Anesthesia for Endovascular Treatment of Acute Ischemic Stroke in Posterior Circulation (CANVAS II) trial suggested conscious sedation was not better than GA in posterior circulation acute ischemic stroke [22].

Despite the similar results of primary outcome between the two treatment arms, the use of general anesthesia showed higher successful recanalization rates in GOLIATH and GASS trials and better functional outcomes at 90 days in GOLIATH [23]. SAGA meta-analysis suggested patients who received general anesthesia have a better functional outcome at 3 months and a higher successful recanalization rate [10]. In our study, the successful recanalization rate was similar between the two groups, while GA tended to be associated with a nominally lower severe procedural complication rate, possibly due to the more convenient operating condition under GA.

However, the results of subgroup analysis and registry population analysis from the Multicenter Randomized Clinical Trial of Endovascular Treatment for Acute Ischemic Stroke in the Netherlands (MR CLEAN) and the Interventional Management of Stroke (IMS) III trial demonstrated higher rates of death or disability with GA [8, 24–26]. Our study found that early neurological improvement was better in non-GA group than in GA group. The systematic review by the highly effective reperfusion using multiple endovascular devices (HERMES) collaboration and Italian Registry of Endovascular Treatment in Acute Stroke (IRETAS) study also reported higher rates of death or disability with GA [9, 27]. The discrepancy is partially caused by non-randomized selection, which may lead to unavoidable bias and have a confounding impact on the subgroup analysis. In the previous studies favored GA, the time window in the target population was different due to the inclusion criteria of stroke onset for alteplase use (6- and 3-h). Also, the existence of diversified treatment in MR CLEAN and lower successful recanalization rate also made the results confounded.

Overall, the present findings of RCTs plus the meta-analysis indicate a similar clinical benefit between the two approaches. The patients in GA group might have lower ASPECTS scores with more severe neurological deficits and longer time intervals from randomization to perfusion. In this study, the bias of lower ASPECTS and workflow delay in GA group was also observed, which reversely boosts the benefit of non-GA approach. In contrast, severe manipulation complication was a major concern for patients who received mechanical thrombectomy under non-general anesthesia. Patient movement due to decreased consciousness level or pain caused by retrieval of stent retrievers could induce manipulation mistakes, possibly leading to vessel perforation and perforator arteries rupture caused by sudden artery displacement. The findings from HERMES meta-analysis showed there was no significant difference in the rate of vessel perforation between patients who had GA or non-GA [9]. In our study, the rate of contrast extravasation on dynamic angiogram confirmed by the core laboratory team tended to be higher in non-GA group, leading to a high mortality rate of 42.9% (6/14) in the complicated cases.

Efficient workflow leads to improved functional outcomes among patients who underwent mechanical thrombectomy [28]. In this study, randomization to reperfusion time in GA group was significantly delayed for 23 min, mainly attributed to the additional general anesthesia procedures. Even though, the prolonged workflow interval did not directly lead to a worse functional outcome, suggesting multiple factors affected the influence of anesthesia. In the DIRECT-MT trial, general anesthesia was mostly performed by a separate team which needs response time to arrive in angiosuite and sign wrote consent before intubation. Fast anesthetic induction and intubation were not common in most participating centers. Compared with the median time interval reported by 7 RCTs enrolled in HERMES meta-analysis, the median time from randomization to reperfusion is longer about 13 min in this subgroup study [9]. Meanwhile, local anesthesia was more commonly used instead of conscious sedation because the anesthesiologist who came from a separate team was mostly in charge of GA, not conscious sedation. In many emergent cases, the performance of local anesthesia for non-GA patients was conducted by operators. The surgical outcome can also be influenced by insufficient intra-operational monitoring (such as blood pressure) and patients’ sedation control. Therefore, a dedicated anesthesia team with streamlined protocols probably enhances anesthesia efficiency instead of a separate anesthesia team.

There are several limitations in this subgroup study. First, unbalanced baseline characteristics confound the analysis of clinical outcomes because the patient selection was not randomized for GA. The important prognostic variable of baseline ASPECTS and time from onset to randomization favored the non-GA group. Although multivariable ordinal regression was used to adjust the confounders, the bias was difficult to eliminate. Second, the differences between anesthesia and conscious sedation in non-GA group were not investigated, because local details pertaining to conscious sedation or local anesthesia including agents used and hemodynamic parameters during the MT were not recorded. The in-depth analysis of the efficiency of anesthesia modality was limited. Third, the judgment of the final results was affected by the intention-to-treat principle. A portion of patients who were converted from non-GA to GA during thrombectomy was scored as non-GA group. In addition, anesthesia has been performed if patients receiving catheter angiography, but some might withdraw from thrombectomy because of the operational complication at the beginning of thrombectomy. These patients were all included as intention-to-treat patients.

In conclusion, these findings in DIRECT-MT showed no significant difference in functional outcome between GA and non-GA groups for patients who received mechanical thrombectomy. The results were consistent with that from recent RCTs on anesthesia, indicating patients derive similar benefits from general anesthesia. The rate of severe manipulation-related complications tends to be higher in non-GA group while the workflow time interval was significantly delayed in GA group. These findings imply efficient workflow for general anesthesia and strict anesthesia protocol for patient movement for patients with non-GA are warranted to improve clinical outcomes.

## Supplementary Information

**Additional file 1: Figure S1.** The adjusted logistic regression for the functional outcome between general anesthesia and non-general anesthesia group. CI, confidence interval; eTICI, extended thrombolysis in cerebral infarction score; mRS, modified Rankin Scale; OR, odds ratio. **Figure S2.** Logistic regression for the difference in safety outcomes and technical complications between the two treatment groups. Non-GA, non-General Anesthesia; GA, General Anesthesia.

## Data Availability

The data that support the findings of this study are available from the corresponding author upon reasonable request.
