# Supplementary material for: Impact of anesthesia modalities on functional outcome of mechanical thrombectomy in patients with acute ischemic stroke: a subgroup analysis of DIRECT-MT trial
Source: Eur J Med Res. 2023 Jul 10;28:228. doi: 10.1186/s40001-023-01171-x (PMC10332003; doi:10.1186/s40001-023-01171-x)
Supplement: Supplementary file 1 — Additional file 1: Figure S1. The adjusted logistic regression for the functional outcome between general anesthesia and non-general anesthesia group. CI, confidence interval; eTICI, extended thrombolysis in cerebral infarction score; mRS, modified Rankin Scale; OR, odds ratio. Figure S2. Logistic regression for the difference in safety outcomes and technical complications between the two treatment groups. Non-GA, non-General Anesthesia; GA, General Anesthesia. [file 40001_2023_1171_MOESM1_ESM.pdf]

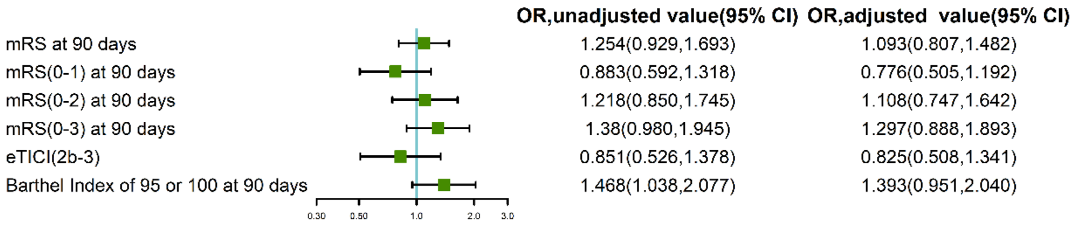

eFigure 1 The adjusted logistic regression for the functional outcome between general anesthesia and non-general anesthesia group

CI, confidence interval; eTICI, extended thrombolysis in cerebral infarction score; mRS, modified Rankin Scale; OR, odds ratio;

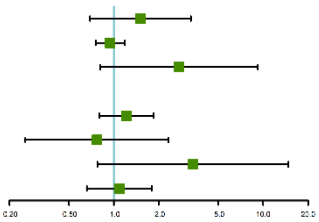

	Non-GA	GA	Difference	Risk ratio(95% CI)	P
**Safety outcome**					
Symptomatic Intracranial hemorrhage	25(5.83%)	8(3.86%)	1.97%	1.508(0.692,3.285)	0.296
Asymptomatic intracranial hemorrhage	151(35.20%)	77(37.20%)	-2.00%	0.946(0.760,1.178)	0.622
Infarction in new territory at 5–7 days	17(3.96%)	3(1.45%)	2.51%	2.734(0.810,9.226)	0.089
**Technical complications**					
Any procedural complications	68(15.85%)	27(13.04%)	2.81%	1.215(0.803,1.838)	0.352
Vessel dissection	8(1.86%)	5(2.42%)	-0.56%	0.772(0.256,2.331)	0.872
Contrast extravasation	14(3.26%)	2(0.97%)	2.29%	3.378(0.775,14.724)	0.083
Embolization into a new territory	45(10.49%)	20(9.66%)	0.83%	1.086(0.659,1.790)	0.747

eFigure 2 Logistic regression for the difference in safety outcomes and technical complications between the two treatment groups

Non-GA, non-General Anesthesia; GA, General Anesthesia;
